# Circulating levels of neurofilament light chain as a biomarker of infarct and white matter hyperintensity volumes after ischemic stroke

**DOI:** 10.1038/s41598-024-67232-1

**Published:** 2024-07-13

**Authors:** Lukas Holmegaard, Christer Jensen, Annie Pedersen, Christian Blomstrand, Kaj Blennow, Henrik Zetterberg, Katarina Jood, Christina Jern

**Affiliations:** 1 Department of Clinical Neuroscience, Institute of Neuroscience and Physiology, Sahlgrenska Academy, University of Gothenburg, Gothenburg, Sweden; 2 Department of Neurology, Sahlgrenska University Hospital, Region Västra Götaland, Gothenburg, Sweden; 3 Department of Radiology, Institute of Clinical Sciences, Sahlgrenska Academy, University of Gothenburg, Gothenburg, Sweden; 4 Department of Radiology, Sahlgrenska University Hospital, Region Västra Götaland, Gothenburg, Sweden; 5 Department of Laboratory Medicine, Institute of Biomedicine, Sahlgrenska Academy, University of Gothenburg, Gothenburg, Sweden; 6 Department of Clinical Genetics and Genomics, Sahlgrenska University Hospital, Region Västra Götaland, Gothenburg, Sweden; 7 Department of Psychiatry and Neurochemistry, Institute of Neuroscience and Physiology, Sahlgrenska Academy, University of Gothenburg, Gothenburg, Sweden; 8 Clinical Neurochemistry Laboratory, Sahlgrenska University Hospital, Mölndal, Region Västra Götaland, Gothenburg, Sweden; 9 Department of Neurodegenerative Disease, UCL Institute of Neurology, Queen Square, London, UK; 10 UK Dementia Research Institute at UCL, London, UK; 11 Hong Kong Center for Neurodegenerative Diseases, Hong Kong, China

**Keywords:** Stroke, Stroke, White matter disease

## Abstract

Serum neurofilament light chain protein (sNfL) shows promise as a biomarker for infarct size in acute ischemic stroke and for monitoring cerebral small vessel disease (cSVD). However, distinguishing the cSVD contribution after stroke may not be possible due to post-stroke sNfL increase. Additionally, it remains unclear if etiologic subtype differences exist. We measured infarct and white matter hyperintensity (WMH) volumes using MRI at the index stroke in ischemic stroke patients (n = 316, mean age 53 years, 65% males) and at 7-year follow-up (n = 187). Serum NfL concentration was measured in the acute phase (n = 235), at 3-months (n = 288), and 7-years (n = 190) post stroke. In multivariable regression, acute and 3-month sNfL concentrations were associated with infarct volume and time since stroke, but not with stroke etiology or infarct location. Seven years post-stroke, sNfL was associated with WMHs and age, but not with stroke etiology. Nonlinear regression estimated that sNfL peaks around 1 month, and declines by 50% at 3 months, and 99% at 9 months. We conclude that sNfL can indicate infarct volume and time since brain injury in the acute and subacute phases after stroke. Due to the significant post-stroke sNfL increase, several months are needed for reliable assessment of cSVD activity.

## Introduction

Neurofilament light chain (NfL) is a marker of neuroaxonal damage^1^. While initial studies focused on measuring NfL in cerebrospinal fluid, advancements in ultrasensitive detection technologies have enabled reliable quantifications also in blood. Serum NfL (sNfL) shows promise as a blood biomarker for monitoring neurodegenerative and neuroinflammatory diseases like Alzheimer’s disease and multiple sclerosis, and possible also for cerebral small vessel disease (cSVD)^2,3^. Several studies have reported an association of increased sNfL with magnetic resonance imaging (MRI) findings of lacunar infarcts^4,5^ and white matter hyperintensities (WMHs) of presumed vascular origin^2,6–9^. However, in ischemic stroke patients, the use of sNfL for monitoring cSVD is complicated by the fact that after an acute cerebrovascular event there is a substantial increase in sNfL^2,6,10,11^, and the time required after this event for sNfL to accurately reflect activity in diffuse cSVD, i.e. manifestations of cSVD except for focal infarcts, is unclear.

Information on the temporal pattern of sNfL after ischemic stroke is scarce, but serum concentrations seem to peak somewhere between 7 days and 3 months^2,6,10,12,13^, and to return to pre-stroke concentrations somewhere between 6 and 15 months^2,6,10,12,14^. In a study of sNfL concentrations after limited white matter axonal injuries evoked by neurosurgical ventricular catheter insertion, it was found that the sNfL concentration peaked at 1 month and returned to baseline after 6–9 months^15^. Additionally, the relevance of differences between etiologic stroke types in this context is yet to be determined. Addressing these knowledge gaps is crucial, as sNfL holds potential as a biomarker for both determining infarct size in ischemic stroke^2,6,10,11^ and for monitoring cSVD activity^2,3^ post-stroke.

Temporal profiles of sNfL after neurosurgical induced white matter axonal injuries, appear to adhere to first-order kinetics in the release and elimination of NfL^15^. We hypothesized that the temporal profile of sNfL after ischemic stroke would exhibit a similar pattern and be proportional to the infarct size and thereby possible to describe mathematically. Further we hypothesized that after the effects of the acute infarct has subsided, sNfL will reflect the progression rate of WMHs as a marker of diffuse cSVD, regardless of the etiology of the index ischemic stroke. In summary, we propose that sNfL concentrations can be used as a biomarker for both acute and chronic progressive damage resulting from an ischemic stroke and diffuse cSVD, respectively, but during different time periods.

## Methods

### Study population

The study sample consisted of participants from the prospective Sahlgrenska Academy Study on Ischemic Stroke (SAHLSIS)^16^, Magnetic Resonance Imaging (MRI) substudy^17^ excluding four subjects for whom the digital MRI images had insufficient quality (n = 316). Patients aged 18–69 years with first-ever or recurrent ischemic stroke were consecutively recruited at the stroke unit at the Sahlgrenska University hospital from August 1998 to December 2004 and were examined in the acute phase and at a follow-up visit after 3 months (late subacute phase). Ischemic strokes were classified into etiologic subtypes, neurological deficits (stroke severity) were assessed, and information on vascular risk factors and neurological comorbidities that may affect sNfL concentrations were acquired as described in the online supplemental information. Surviving participants were invited to participate in a 7-year follow-up, including a re-examination of the brain with MRI and repeated blood sampling. Information on neurological comorbidities were acquired through questionnaires distributed to all participants at baseline and at the 7-year follow-up, and through searching the Swedish Hospital Discharge register for the total follow-up period for all participants for ICD-10 codes G10-G14 (systemic atrophies primarily affecting the central nervous system), G20-G26 (extrapyramidal and movement disorders), G30-G32 (other degenerative diseases of the nervous system), and G35-G37 (demyelinating diseases of the central nervous system). From this information, participants with neurological diseases with a possible influence on the sNfL concentration were identified.

### Neuroimaging

The MRI examinations at the index stroke were performed on MRI scanners used in clinical routine. Proton density (PD), T1-, and T2-weighted sequences were acquired, and in some cases, depending on the clinical context and the scanner available, additional sequences were also included^17^. At the 7-year follow-up MRI, the study protocol included T1- and T2-weighted sequences, fluid-attenuated inversion recovery (FLAIR) sequences, diffusion-weighted images (DWI), and apparent diffusion coefficient (ADC) maps^17^.

All MRI examinations were reviewed and assessed by a single neuroradiologist (C. Jensen). Quantitative measurements of infarct and WMH volumes were then performed by a single neurologist (L.H.). Infarcts were manually delineated, and volumes were calculated using 3D Slicer version 3.10.2 (National Alliance for Medical Image Computing, USA). Since the MRI examinations in the acute and subacute phases were made at different time points, information from all available sequences was utilized to make the infarct delineation.

The same method was used for WMHs, appearing as hyperintensities on T2-, PD-, and FLAIR-sequences. Here, the volume of WMHs on the side without infarcts, or in cases of bilateral lesions, the volume on the side that was least affected by infarcts, was determined and then doubled to estimate the total WMH burden. WMH progression during the 7-year follow-up period was determined as the change in WMH volume from the index stroke to the 7-year follow-up. Since the focus of our study was to investigate the association between lesion volumes and the concentration of sNfL, lesion volumes were not adjusted for total brain volume.

For determining infarct location, a map of the brain including 51 anatomical regions on each side of the midline was used. A single neuroradiologist (C. Jensen) manually assessed whether an infarct was present or not in each region. See Table S1 in the online supplement for details.

Further, for characterizing the patients at the index stroke, WMHs of presumed vascular origin, were also assessed using a visual rating scale based on Fazekas’ classification system^18^, as described in detail elsewhere^17^.

### Blood sampling and measurements of sNfL

Blood samples were drawn during the hospital stay (acute phase) and at the 3-month (late subacute phase) and 7-year follow-ups. On all occasions, venous blood was collected between 8.30 and 10.30 a.m. after an overnight fast. Serum was isolated within two hours by centrifugation at 2000×*g* at 4 °C for 20 min. All samples were aliquoted and stored at − 80 °C. Serum NfL concentration was measured using an in-house assay on the Single molecule array (Simoa) platform (Quanterix, Billerica, MA), as previously described^19^. All analyses were performed by board-certified laboratory technicians who were blind to clinical information. The three samples from each patient were analyzed on the same occasion.

### The time course of sNfL after acute ischemic stroke

We calculated the time course of changes in sNfL concentrations after the ischemic event based on first-order kinetics for the release and elimination of NfL. The rationale behind this is an observation that the time curves of sNfL after limited white matter axonal injuries evoked by neurosurgical ventricular catheter insertion^15^ seems to follow such curves. The concentration of sNfL as a function of time and infarct volume could then be described with the following expression:

1 $$C_{NfL} \, = \, C_{0} \, + \, k_{0} V_{0} \, \left( {e^{{ - \frac{\ln 2}{{t_{e} }}t}} - e^{{ - \frac{\ln 2}{{t_{r} }}t}} } \right)$$

where *C*_*NfL*_ is the serum concentration [pg/ml], *V*_*0*_ is the infarct volume [ml], *t* is the time since the stroke [days], *C*_*0*_ is the concentration at time zero [pg/ml], *k*_*0*_ is a kinetic constant [pg/ml/cm^3^], *t*_*e*_ is the half-life for elimination [days], and *t*_*r*_ is the half-life for release [days].

### Statistical analyses

For patient characteristics at the index stroke, differences between groups were evaluated using χ^2^-tests for proportions and Mann–Whitney U-tests for numerical variables.

Correlations between sNfL concentrations and infarct/WMH volumes at different time points were determined using Spearman's rank correlation coefficient. Since patients were recruited after the acute ischemic stroke we did not have access to the sNfL concentration before the ischemic event. Because we expected that the contribution to sNfL from the acute ischemic lesion would obscure the smaller contribution from diffuse cSVD, we performed two pre-specified subgroup analyses of correlations. These analyses aimed to investigate if we could find an association between sNfL and WMH in cases with early blood sampling (defined as within the first 3 days after index stroke), presumably before sNfL concentrations due to the acute infarct had risen too high, and in cases with small infarcts (defined as < 2.5 cm^3^), presumedly with a limited contribution to sNfL.

For the nonlinear model of sNfL concentration in the acute and subacute phases (Eq. 1), parameters were determined using non-linear least squares regression with the Nash variant of the Marquardt algorithm^20^. Since the algorithm does not guarantee finding a global minimum in cases of multiple minima, the results were later verified by systematically testing all possible combinations of parameter values in steps of 0.1 within their possible ranges to find the combination that minimized the sum of squared residuals. The lengths of time until maximum increase in sNfL, a 50% decline, and a 99% decline were then calculated from the model. Confidence intervals for these time points were subsequently calculated by bootstrapping the modeling process, except for checking for global minima in each bootstrap step, which was precluded due to time constraints. Finally, the model was evaluated using the half of the participants not used for model construction.

To investigate the influence of factors other than infarct volume and time on sNfL levels in the acute and subacute phases, as predicted by the nonlinear model, we performed multivariable mixed model analyses with patient ID as a random factor. In these analyses, sNfL predicted from Eq. 1 was included instead of directly incorporating infarct volume and time since the stroke, to accomplish a linearization of the association. Here we expected, that if the concentrations of sNfL predicted from infarct volume and time indeed reflected the actual concentrations, the increase in sNfL for a unit increase in predicted sNfL would be close to one. In the first model, we included predicted sNfL, age, sex, vascular risk factors, etiologic subtype, and WMH volume at baseline. In the second model, we examined the influence of infarct location by adding a variable for infarct location along with the significant variables from the first model. In the third model, we investigated whether differences between etiologic subtypes found in the second model could be explained by some infarcts being small, by including a variable for infarct size being less than 2.5 cm^3^. In the fourth model, only the significant variables from the third model were retained.

To investigate the association between cSVD activity, using WMH progression as a proxy, and sNfL, we employed generalized linear modeling, accounting for age, sex, vascular risk factors, and etiologic stroke type. We first calculated univariate associations and then included the significant variables from these analyses in a multivariable model. The significant variables from this first multivariable model were then used in a second model. In a third model, we replaced the variable for WMH progression with WMH volume at 7 years, and in a fourth model, we replaced the variable with WMH volume in the acute phase.

Statistical calculations were performed in *R* version 4.0.3 (R Foundation for Statistical Computing, Vienna, Austria) with the packages: *boot* version 1.3.25, *nlmrt* version 2016.3.2, and *lme4* version 1.1.26, and in Matlab version R2021a (MathWorks, Natick, MA).

### Ethical approval

The study was approved by the Regional Ethics Review Board in Gothenburg, Sweden (469-99, T553-03, 413-04, T665-07) and performed in accordance with the 1964 Helsinki Declaration. Written informed consent was obtained from all participants or from their next of kin prior to enrollment. For participants who were unable to communicate, consent was obtained from their next-of-kin.

## Results

Baseline characteristics of the study population are presented in Table 1. The study included 316 patients with a mean age of 53 years, of which 65% were males. The majority of the cases experienced a mild stroke, had small infarcts and small WMH volumes, but values ranged up to an NIHSS score of 22 p, an infarct volume of 357 cm^3^, and a WMH volume of 47 cm^3^. Compared to subjects who had an MRI at the 7-year follow-up, those with an MRI only at the time of the index stroke had larger infarct and WMH volumes, more neurological deficits, higher sNfL concentrations, and a higher proportion of cardioembolic strokes. Compared to patients experiencing their first-ever stroke (n = 265), those with a history of previous stroke (n = 51) were older, had a higher frequency of hypertension, and showed a greater WMH burden. They also more frequently exhibited a combination of supra- and infratentorial infarcts and were more often diagnosed with the etiologic subtype of large artery atherosclerosis. In contrast, cervical artery dissection and cryptogenic stroke were less common in this group. However, there were no differences in acute infarct volume and sNfL concentration between the two groups. Please refer to Table S2 in the supplement for details. At baseline, one participant had a comorbidity (Alzheimer’s disease) which could have affected sNfL, but was still included in the study. At the 7-year follow-up, the participant with Alzheimer’s disease was deceased, and none of the other participants had developed any neurological comorbidity known to affect sNfL. Data availability at the different time points and reasons for missing data are shown in Table 2.

**Table 1:** Patient characteristics at the index stroke.

	Overall	Subject with MRI at 7 years	Subjects without MRI at 7 years
	(n = 316)	(n = 187)	(n = 129)
Age in years, mean (SD)	53 (11)	53 (11)	54 (12)
Male sex, no. (%)	204 (65)	122 (65)	82 (64)
Hypertension, no. (%)	171 (54)	102 (55)	69 (53)
Diabetes mellitus, no. (%)	54 (17)	27 (14)	27 (21)
Hyperlipidemia, no. (%)	215 (68)	129 (69)	86 (67)
Smoking, no. (%)	123 (39)	66 (35)	57 (44)
Subtype—Cryptogenic, no. (%)	110 (35)	71 (38)	39 (30)
- Small artery occlusion, no. (%)	55 (17)	36 (19)	19 (15)
- Large artery atherosclerosis, no. (%)	46 (15)	22 (12)	24 (19)
- Cardioembolic, no. (%)	38 (12)	16 (9)	22 (17)*
- Cervical artery dissection, no. (%)	29 (9)	19 (10)	10 (8)
- Other determined, no. (%)	13 (4)	7 (4)	6 (5)
-Undetermined, no. (%)	25 (8)	16 (9)	9 (7)
SSS, M (IQR)	54 (43 to 56)	54 (46 to 57)	51 (32 to 55)**
NIHSS (estimated), M (IQR)	2 (1 to 6)	2 (1 to 5)	3 (2 to 11)**
Infarct location—Supratentorial cortical, no. (%)	129 (41)	70 (37)	59 (46)
- Supratentorial non cortical, no. (%)	87 (28)	51 (27)	36 (28)
- Infratentorial, no. (%)	46 (15)	32 (17)	14 (11)
- Supra and infratentorial, no. (%)	28 (9)	21 (11)	7 (5)
- Undetermined, no. (%)	26 (8)	13 (7)	13 (10)
Small infarcts (< 2.5 cm^3^), no. (%)	144 (46)	98 (52)	46 (36)
Fazekas—Deep WMHs > 1, no. (%)	57 (18)	30 (16)	27 (21)
- Periventricular WMHs > 1, no. (%)	38 (12)	22 (12)	16 (12)
Infarct vol. [cm^3^], mean (SD)	27.9 (54.5)	20.7 (45.3)	38.4 (64.4)**
WMH vol. [cm^3^], mean (SD)	2.9 (6.5)	2.6 (6.3)	3.4 (6.8)*
Serum NfL [pg/ml], mean (SD)	177 (325)	135 (229)	240 (423)*

IQR = interquartile range; M = median; MRI = magnetic resonance imaging; NIHSS = National Institutes of Health Stroke Scale; SD = standard deviation; SSS = Scandinavian Stroke Scale; WMH = white matter hyperintensity. *P < 0.05; **P < 0.01.

**Table 2:** Data availability at different time points.

	Participants	Missing
Acute phase and 3-months	316	
- MRI	316	
- sNfL acute	235	Before Oct. 1999, serum was not collected in the acute phase (64); serum not available or not analyzable (17)
- sNfL 3-months	288	Deceased (7); serum not available or not analyzable (21)
7-year follow-up	193	Deceased (28); declined (33); severe disability (25); lost to follow up (15); severe illness (9); long distance (4); not in Sweden (2)
- MRI	187	Contraindications (6)
- sNfL	190	Serum not available or not analyzable (3)

MRI = magnetic resonance imaging; sNfL = Serum neurofilament light chain protein.

### Neuroimaging and sNfL in the acute and subacute phases after ischemic stroke

The median time to the first MRI scan was 8 days (IQR 4–75). Concentrations of sNfL were determined in 235 and 288 stroke cases in the acute phase and at 3 months, respectively, please see Table 2. The mean concentrations of sNfL in the acute and the late subacute phases were 177 pg/ml (95% CI 138–221) and 185 pg/ml (95% CI 155–217), respectively.

#### Correlations between sNfL and infarct and WMH volumes

**Figure 1:**
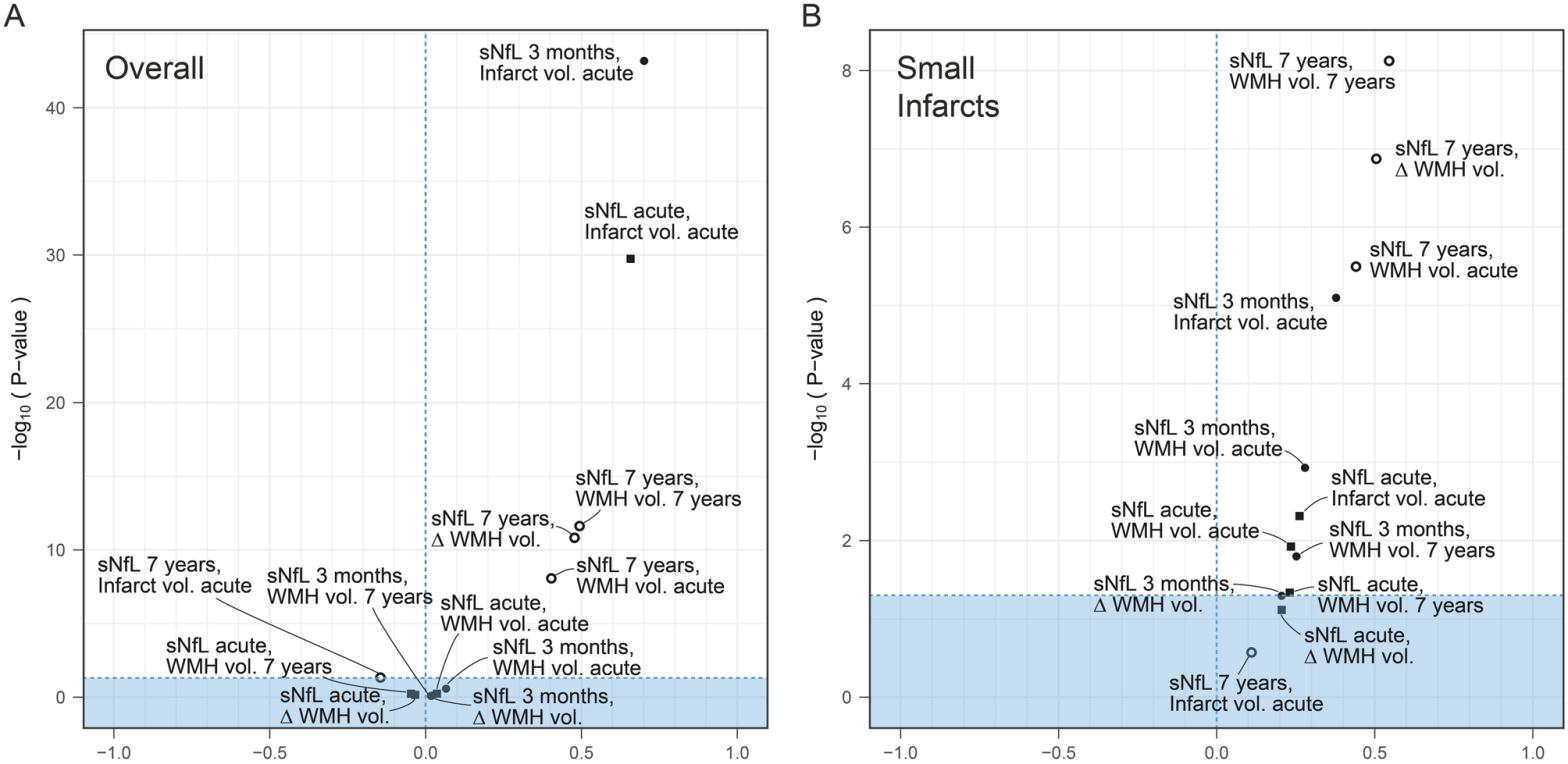
Volcano plots of correlations between sNfL concentrations and infarct/WMH volumes in (**A**) the overall group, and in (**B**) a subgroup analysis including only subject with small infarcts (< 2.5 cm^3^). Points in the plots indicate pairwise Spearman's rank correlations coefficients and the associated *P* values. Shaded areas represent *P* > 0.05. Δ = change from index stroke to 7-year follow-up; sNfL = serum neurofilament light chain protein; WMH = white matter hyperintensity.

Correlations between sNfL and infarct and WMH volumes at the different time points are shown in Fig. 1A. For exact numerical values, please refer to Table S3A in the online supplement. Serum NfL in the acute- and late subacute phases showed strong correlations with infarct volume, but no correlations were observed with WMH volumes.

In the first subgroup analysis of patients whose blood samples were obtained within the first 3 days after index stroke (n = 68), no significant correlations between sNfL and WMH volumes were found, please refer to Figure S1 in the online supplement for detailed results.

In the second subgroup analysis of patients with small infarcts (< 2.5 cm^3^), sNfL both in the acute and late subacute phases were significantly correlated with the WMH volume both in the acute phase and at the 7-year follow-up (Fig. 1B). For exact numerical values, please refer to Table S3B in the online supplement.

#### Time course of sNfL after acute ischemic stroke

**Figure 2:**
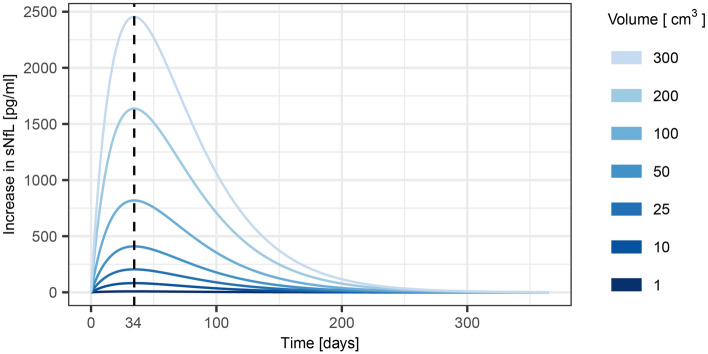
Estimated increases in sNfL concentrations after ischemic stroke with infarcts of different volumes. sNfL, serum neurofilament light chain protein.

**Figure 3:**
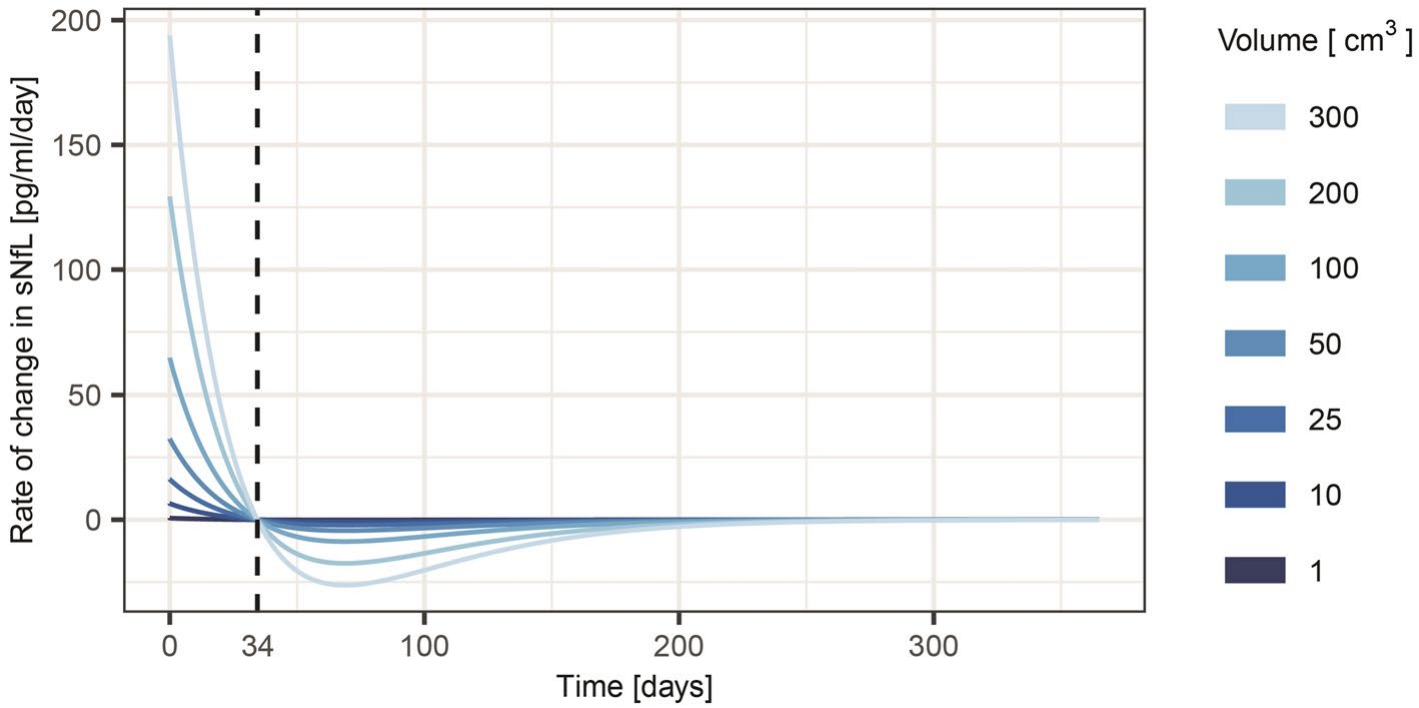
Estimated rates of change in sNfL concentrations after ischemic stroke with infarcts of different volumes. sNfL, serum neurofilament light chain protein.

For the time course of sNfL after acute ischemic stroke, the nonlinear regression of sNfL on infarct volume and time since the index stroke yielded the following parameter values for Eq. 1: *C*_*0*_ = 89.2 pg/ml*, k*_*0*_ = 477.4 pg/ml/cm^3^, *t*_*e*_ = 24.4 days, and *t*_*r*_ = 23.3 days. Inserting these parameters into Eq. 1 yields the following expression for the sNfL concentration:

2 $$C_{NfL} { = 89}{\text{.2 }} + \, 477.4 \, V_{0} \, \left( {e^{{ - \frac{\ln 2}{{24.4}}t}} - e^{{ - \frac{\ln 2}{{23.3}}t}} } \right)$$

where* V*_*0*_ is the infarct volume, and *t* is the time since the stroke. The increase in sNfL concentration and its rate of change following an ischemic stroke with infarcts of varying volumes predicted by the model is displayed in Figs. 2 and 3. Time to the maximum increase in sNfL was estimated to be 34 days (95% CI 26–49), and the times to when the increase had declined with 50% and with 99% were estimated to be 92 days (95% CI 71–134), and 263 days (95% CI 207–383), respectively. Model evaluation, using 50% of the participants not included in model fitting, showed that the correlation between the predicted and observed values was strong with a Spearman’s ρ of 0.71 (*P* < 3 × 10^–16^). An illustration of the model with the observed data points from all participants overlayed, can be viewed in Figure S2 in the online supplement.

The results from the multivariable mixed model analyses, which aimed to investigate the influence of additional factors on sNfL concentration beyond the predicted values, are presented in Table 3. As expected, if the concentrations of sNfL predicted from infarct volume and time indeed reflected the actual concentrations, the increase in sNfL for a unit increase in predicted sNfL was close to one.

**Table 3:** Regression analyses results for sNfL concentrations during the acute and late subacute phases.

	Model 1	Model 2	Model 3	Model 4
Predicted sNfL, per pg/ml	1.1 (0.9 to 1.2) ***	1.0 (0.8 to 1.1)***	1.0 (0.8 to 1.1)***	1.0 (0.8 to 1.1)***
Age, per year	0.5 (− 1.8 to 2.8)	N.I	N.I	N.I
Male sex	− 45.0 (− 89.8 to − 0.1)*	− 48.8 (− 94.5 to − 3.2)*	− 48.3 (− 93.1 to − 3.4)*	− 42.0 (− 87.2 to 3.2)
Hypertension	16.5 (− 29.5 to 62.5)	N.I	N.I	N.I
Hyperlipidemia	26.7 (− 23.9 to 77.3)	N.I	N.I	N.I
Diabetes mellitus	14.8 (− 44.7 to 74.0)	N.I	N.I	N.I
Smoking	− 3.2 (− 47.4 to 41.1)	N.I	N.I	N.I
Etiologic subtype				
CE	0 (reference)	0 (reference)	0 (reference)	
LVD	6.5 (− 78.1 to 91.3)	18.1 (− 65.9 to 102.4)	21.3 (− 62.2 to 105.0)	N.I
CAD	− 17.8 (− 112.6 to 77.2)	− 0.1 (− 98.9 to 99.0)	− 29.4 (− 124.1 to 65.6)	N.I
SAO	− 115.5 (− 197.9 to − 33.0)**	− 94.5 (− 182.9 to − 5.9)*	− 65.5 (− 153.7 to 22.9)	N.I
Other determined	− 96.7 (− 216.0 to 23.1)	− 91.9 (− 213.9 to 30.7)	− 86.8 (− 207.9 to 34.8)	N.I
Cryptogenic	− 72.0 (− 144.5 to 0.4)	− 72.4 (− 145.5 to 1.0)	− 65.0 (− 138.4 to 8.6)	N.I
Undetermined	− 81.6 (− 178.3 to 15.2)	− 56.2 (− 154.6 to 42.3)	− 52.7 (− 151.0 to 45.6)	N.I
WMH vol., per cm^3^	2.7 (− 0.8 to 6.2)	N.I	N.I	N.I
Infarct location				
Supratent., cortex affected	N.I	0 (reference)	N.I	N.I
Supratent., non-cortical	N.I	− 24.8 (− 86.2 to 36.5)	N.I	N.I
Infratentorial	N.I	− 68.0 (− 138.7 to 2.5)	N.I	N.I
Supra- and infratentorial	N.I	− 8.8 (− 90.8 to 73.2)	N.I	N.I
No visible infarct	N.I	− 88.6 (− 174.5 to − 2.8)*	N.I	N.I
Small infarct (< 2.5 cm^3^)	N.I	N.I	− 71.3 (− 123.9 to − 18.9)**	− 93.0 (− 140.8 to − 45.3)***
R^2^ for model	0.57	0.56	0.56	0.56
R^2^ for fixed effects	0.42	0.41	0.41	0.39

Mixed effects models with patient ID as random factor. Values are changes in sNfL [pg/ml] associated with various characteristics, and with unit increases in continuous variables (i.e. β-coefficients). Within parentheses are 95% confidence intervals. Predicted sNfL concentration was determined from infarct volume and time since the index stroke, in accordance with Eq. 2 described in the present paper. CAD, cervical artery dissection; CE = cardioembolism; LAA = large artery atherosclerosis; NfL = neurofilament light chain protein; N.I. = not included in model; SAO = small artery occlusion; sNfL = serum NfL; WMH = white matter hyperintensity. *P < 0.05; **P < 0.01; ***P < 0.001.

In the first model, it was found that, in addition to predicted sNfL, male sex was associated with lower concentrations of sNfL. Additionally, the concentration of sNfL was significantly lower in small artery occlusion (SAO) stroke compared to cardioembolic (CE) stroke. A closer analysis of differences between subtypes, performed by varying the subtype used as the reference in the regression analysis and applying Bonferroni adjustments, showed that the concentration of sNfL in SAO was also significantly lower than in large artery atherosclerosis (LAA) stroke. No other significant differences between subtypes were found. Furthermore, there were no significant associations with age or vascular risk factors.

In the second model, which included a variable indicating infarct location along with the significant variables from the first model we found that those with no visible infarcts differed, but no significant differences between infarct locations were found. As in the first model, male sex was associated with lower concentrations of sNfL, and the concentration of sNfL was significantly lower in small artery occlusion (SAO) stroke compared to cardioembolic (CE) stroke.

In the third model, we investigated whether differences between etiologic subtypes found in the second model could be explained by some infarcts being small. A variable indicating small infarcts was included along with the significant variables from the second model. In this model, the variable indicating small infarcts was significant, whereas no significant differences between etiologic subtypes remained.

In the fourth model, which included the significant variables from the third model, we found that, in addition to predicted sNfL, only the variable for small infarcts remained significant.

### Neuroimaging and sNfL in the long-term after ischemic stroke

Subjects who were alive (n = 281 out of 316) were invited to a 7-year follow-up visit including a repeated MRI scan of the brain and blood sampling. WMH volumes were measured in 187 (67%), and concentrations of sNfL were determined in 190 (68%) of the subjects, please see Table 2. The median time from the index stroke to follow-up was 7.5 years (IQR 7.4–7.6). The mean WMH volume was 9.6 cm^3^ (95% CI 6.8–12.6) with a mean increase in WMH during the follow-up period of 7.0 cm^3^ (95% CI 4.8–9.5). The mean concentration of sNfL was 26 pg/ml (95% CI 23–30).

At the 7-year follow-up, sNfL showed moderate correlations with the increase in WMH volume over the 7-year period, with WMH volume in the acute phase, and with WMH volume at the 7-year follow-up. No correlation was observed with infarct volume, please refer to Fig. 1A.

The results from regression analyses for sNfL concentrations at the 7-year follow-up examining the associations with the progression of WMHs, etiologic subtype of the index stroke, age, sex, and vascular risk factors, are presented in Table 4. In the univariate analyses, sNfL concentration was associated with WMH progression during the follow-up period (R^2^ = 21%), age (R^2^ = 20%), hypertension (R^2^ = 5%), and diabetes mellitus (R^2^ = 3%).

**Table 4:** Regression analyses results for sNfL concentrations at the 7-year follow-up.

	Univariate models	Multivariable model 1
WMH vol. increase, per cm^3^	0.7 (0.5–0.9)***	0.6 (0.4–0.8)***
Age, per year	0.9 (0.7–1.2)***	0.8 (0.5–1.1)***
Male sex	1.5 (− 5.5 to 8.5)	− 0.9 (− 7.2 to 5.3)
Hypertension	9.3 (2.8 to 15.9)**	0.4 (− 5.7 to 6.5)
Hyperlipidemia	5.5 (− 2.0 to 13.1)	− 2.1 (− 9.1 to 4.9)
Diabetes mellitus	10.7 (1.3 to 20.0)*	3.8 (− 4.6 to 12.2)
Smoking	0.0 (− 6.9 to 6.9)	− 0.1 (− 6.2 to 6.0)
Subtype CE (ref)	0 (reference)	0 (reference)
LVD	7.7 (− 7.9 to 23.3)	− 1.4 (− 15.0 to 12.2)
CAD	− 9.0 (− 24.8 to 6.8)	− 2.3 (− 15.8 to 11.2)
SAO	8.8 (− 5.2 to 22.9)	1.5 (− 10.6 to 13.6)
Other determined	10.4 (− 9.9 to 30.7)	14.1 (− 3.3 to 31.5)
Cryptogenic	0.9 (− 12.2 to 14.0)	1.4 (− 9.8 to 12.6)
Undetermined	8.5 (− 7.9 to 24.9)	2.8 (− 11.3 to 16.9)
Estimated R^2^	≤ 21%	38%

Univariate and multivariable regression models. Values are changes in sNfL [pg/ml] associated with various characteristics at the index stroke, unit increases in age, and unit increases in WMH volume during the follow-up period (i.e. β-coefficients). Within parentheses are 95% confidence intervals. Abbreviations as in Table 3.

In the first multivariable model, which included the significant variables from the univariate analyses, we found that only the increase in WMH volume and age remained significant (Table 4). In the second multivariable model, which included the significant variables from model one, both variables remained significant. Specifically, there was an increase in sNfL at 7 years of 0.6 pg/ml (95% CI 0.4–0.8) per cm^3^ increase in WMH volume and an increase of 0.7 pg/ml (95% CI 0.4–1.0) per year increase in age, with R^2^ = 0.32.

In the third multivariable model, which included WMH volume at the 7-year follow-up and age, both variables were significant, with an increase in sNfL at 7 years of 0.5 pg/ml (95% CI 0.4–0.7) per cm^3^ increase in WMH volume and an increase of 0.7 pg/ml (95% CI 0.4–0.9) per year increase in age, with R^2^ = 0.36.

In the fourth multivariable model, which included WMH volume in the acute phase and age, both variables were significant, with an increase in sNfL at 7 years of 1.4 pg/ml (95% CI 1.0–1.9) per cm^3^ increase in WMH volume and an increase of 0.7 pg/ml (95% CI 0.4–1.0) per year increase in age, with R^2^ = 0.34.

Of the 187 participants with MRI at the 7-year follow-up, 24 (13%) had suffered a recurrent stroke during the follow-up period. Repeating the regression analyses for sNfL excluding those who had suffered a recurrent stroke did not change the results in any significant way.

## Discussion

In our study, we found that sNfL concentration during the acute and subacute phases after ischemic stroke was mainly determined by infarct volume and time since stroke. With the exception for the smallest infarcts, the stroke-induced increase in sNfL in these two phases was so extensive that it obscured any contribution of diffuse cSVD. This effect was consistent across etiologies and independent of age and vascular risk factors. However, during long-term follow-up, after the acute stroke influence had declined, we found that sNfL was associated with the progression of WMHs from index stroke and with age, independent of etiology, sex, or the presence of vascular risk factors at the index stroke. Furthermore, our estimations suggest that the influence of the acute stroke on sNfL remains for quite some time. Specifically, our model indicates that sNfL peaks at around 1 month, declines by 50% at 3 months, and by 99% at 9 months after the ischemic event.

The strong influence of cerebral infarct size on sNfL during the first months after ischemic stroke that we found is corroborated by results in previous studies. In the Circulating Biomarkers in Acute Stroke (CIRCULAS) study, a positive correlation was found between infarct volume and sNfL at day 3 and day 7 after index stroke, but not at earlier time points^6^. Another study that measured infarct size based on the maximum sagittal and transverse diameters found associations with sNfL at days 7–9, 3 weeks, and 3–5 months^10^. In a study of small subcortical infarcts, the maximum axial diameter of the infarcts was associated with sNfL within the first 11 days^2^. Another study found a correlation between infarct volumes determined from computed tomography (CT) and sNfL at 3 months^11^. Previous studies have also shown associations between NIHSS score and sNfL within the first days^11^, at 30 days^21^, and at 3 months^12^.

After having adjusted for infarct volume and time, we did not observe any differences in sNfL between etiologic subtypes. This finding provide support for the argument that previously reported differences in sNfL between etiologic subtypes during the first few months post-stroke^11,12^ may be attributed to differences in infarct volume.

Furthermore, we did not observe any significant differences in sNfL concentrations between infarct locations when adjusting for infarct volume and time. Here, one could have expected differences after lesions at different locations due to variation in the cellular architecture and connectivity to remote areas, variation in the vascular supply, and variation in the degree of postischemic Wallerian degeneration^6,22–25^. However, NfL is a non-specific marker of neuro-axonal injury and increased sNfL concentrations have been observed in various conditions that primarily affects different parts of the nervous system such as cortical atrophy in Alzheimer’s disease, atrophy of the basal ganglia, brainstem, and cerebellum in multiple system atrophy, and peripheral nerve injury in Guillain-Barré syndrome^26–29^.

Our estimations of the time to peak concentration of sNfL and its subsequent decline, based solely on assuming the general shape of the curve observed in a previous study on sNfL concentrations following neurosurgically induced white matter damage^15^, but utilizing parameters derived from our data obtained from acute ischemic stroke patients, yielded comparable results to those observed in the referenced study. In that study, sNfL reached its peak at 1 month after surgery and returned to baseline levels within 6–9 months^15^. This alignment between our estimations and the experimental findings supports the relevance and accuracy of our model, while also indicating similarities in sNfL concentrations in acute lesions whether caused by ischemia or trauma. Our estimations are also in line with the results from a study that included repeated measurements of sNfL levels following an ischemic stroke where it was found that concentrations initially increased from day 0–1 to 3 weeks and subsequently, compared with the concentration at 3 weeks, had declined at the next measurement point at 3–5 months^10^. Our use of a nonlinear model allows for a comprehensive description of the entire time course of sNfL after acute ischemic stroke, which is not possible with linear models employed in earlier studies^2,6,12^.

Furthermore, our model of the time course demonstrates that it should be possible to establish whether an acute lesion has occurred or not, as well as obtain an estimate of the size of the lesion, and the time since it occurred, by measuring sNfL at repeated time points. For instance, as illustrated in Figs. 2 and 3, during the first month after ischemic stroke, sNfL is expected to increase with each consecutive day, and larger infarcts would be associated with larger increases per day. During the next few months, sNfL decreases and after approximately 9 months sNfL is estimated to have returned to the value it had before the stroke. Concentrations of sNfL would then be dependent on continuously ongoing subclinical neuronal damage for which cSVD is a frequent cause.

In the overall study population, we found no significant correlation between WMH volume and sNfL in the acute and late subacute phases. Initially, we suspected that during the first days after a stroke, the rise in sNfL caused by the acute infarct would not yet have influenced sNfL levels enough to obscure the contribution of any diffuse cSVD present before the stroke. However a subgroup analysis including only patients whose blood samples were obtained within the first 3 days revealed no such association. Nevertheless, in a subgroup analysis of sNfL in the acute and late subacute phases, including only patients with small infarcts (< 2.5 cm^3^), we found that sNfL, both in the acute and late subacute phases, was significantly correlated with the WMH volume, both in the acute phase and at the 7-year follow-up. Our interpretation is that increased concentration of sNfL is associated with subclinical activity in cSVD, but that the increase in sNfL caused by the stroke is so extensive during the initial period after stroke that it obscures any contribution of diffuse cSVD, except in cases with very small infarcts.

At the long-term follow-up, we found that sNfL 7 years after stroke was associated with an increase in WMHs from the index stroke, with WMH volume at the index stroke and cross-sectionally with WMH volume. The presence of a high degree of WMHs at baseline has been noted in long-term follow-up studies to be the strongest predictor for future progression of WMHs^17,30,31^. Therefore, our findings are expected if sNfL indeed reflects the activity in cSVD. In univariate analyses, hypertension at and diabetes at the index stroke were also associated with sNfL at 7 years, although they explained only a small percentage of the variation. However, in multivariable analysis, these associations were no longer significant. Since these factors have been associated with WMH progression in earlier studies^17,30,31^, their effect on sNfL may at least partly be mediated by their influence on cSVD. Furthermore, we found no significant association between the etiology of the index stroke and sNfL 7 years after stroke when taking age and WMHs into account. Assuming that sNfL reflects the activity of cSVD, this observation is consistent with an earlier study where we did not observe an independent association between the etiology of the index stroke and long-term progression of WMHs^17^. Age has been consistently associated with WMH progression in earlier long-term studies^17,30,31^. However, our finding that age is associated with sNfL even when adjusting for WMHs, indicates that there are additional age-related processes, and possibly neurodegenerative disease processes associated with age, that our study did not detect, which contribute to an increased concentration of sNfL. This is corroborated by population-based studies that show a strong association between sNfL and aging^32,33^.

In the acute and subacute phases, sNfL has the potential to aid in diagnosing or ruling out ischemic stroke. This could be particularly useful in environments with limited access to neuroimaging, and in cases where patients cannot undergo MRI due to contraindications or claustrophobia. In situations where an acute CT scan does not reveal an infarct, repeated measurements of sNfL may replace the need for a confirmatory MRI scan. Additionally, through infarct volume estimations, sNfL could provide prognostic information on functional outcomes^34,35^.

Once the influence of acute ischemic injury has subsided, sNfL could be used to monitor the progression of cSVD as an alternative to repeated MRI scans, which are often impractical, expensive, and inconvenient for patients. Since sNfL measurements are minimally invasive, they can easily be incorporated into clinical practice, including for outpatients. In patients with known cSVD, sNfL can monitor the activity of cSVD over recent time periods and detect the occurrence of covert infarcts or other events causing neuronal injury. Knowing the level of cSVD activity could also provide more accurate prognostic information. The ability to frequently monitor cSVD activity would allow for better evaluation of treatment effects, which could be particularly useful in clinical trials testing candidate drugs.

The current study has several strengths. Firstly, it includes well-characterized patients who were young (< 70 years) at the time of their ischemic stroke, which reduces the potential for confounding comorbidities compared to studies of older stroke patients. Additionally, blood sampling was strictly standardized and samples from the different time points were measured simultaneously using the most sensitive available method^36^. Volumetric measurements from MRI images were performed by the same neurologist, with the assistance of a neuroradiologist throughout the study. This ensures consistency and accuracy, although also increasing the risk of rater bias. Lastly, we utilized a nonlinear model of the sNfL concentration, with the possibility to provide novel information on the kinetics of sNfL in relation to acute infarcts during the acute and subacute phases after stroke. While our study has several important strengths, it also has limitations that should be considered. Firstly, our study was designed to investigate ischemic stroke in individuals under 70 years of age, so our results may not be applicable to older stroke patients with more comorbidities including neurodegenerative diseases. Additionally, the clinical context of the study and the development of MRI technology over the long study period presented several challenges. Consequently, brain MRI scans were conducted using different scanners, with varying field strengths, available image sequences, and image quality. Due to these variations, volumetric measurements were performed manually instead of utilizing automated methods. Another potential limitation is the time lapse between blood sampling and sNfL analysis. However, studies have shown that NfL is stable at − 80 °C and can withstand multiple freeze–thaw cycles^37^.

In conclusion, our study suggests that sNfL can serve as a useful biomarker for infarct size during the acute and subacute phases after ischemic stroke. Repeated measurements during this period may provide information on infarct volume and time since stroke. When the influence of the acute ischemic injury has subsided, which may take up to 9 months according to our present calculations, sNfL is likely to be a useful marker of activity in diffuse cSVD. It is important to note that our study had some limitations, including the restriction to stroke patients younger than 70 years old without neurological comorbidities and with, on average, relatively mild strokes, which may limit the generalizability of our findings to other populations.

### Supplementary Information

Supplementary Information.

## Data Availability

Anonymized data will be shared upon reasonable request from a qualified academic investigator as long as data transfer is in agreement with EU legislation on the general data protection regulation (GDPR) as well as in agreement with decisions by the Ethical Review Board of Sweden and the University of Gothenburg, the latter which should be regulated in a data transfer agreement.
