# Supplementary material for: Circulating levels of neurofilament light chain as a biomarker of infarct and white matter hyperintensity volumes after ischemic stroke
Source: Sci Rep. 2024 Jul 13;14:16180. doi: 10.1038/s41598-024-67232-1 (PMC11246414; doi:10.1038/s41598-024-67232-1)
Supplement: Supplementary file 1 — Supplementary Information. [file 41598_2024_67232_MOESM1_ESM.pdf]

Supplementary information to

Circulating Levels of Neurofilament Light Chain as a Biomarker of Infarct and White Matter Hyperintensity Volumes After Ischemic Stroke

Lukas Holmegaard; Christer Jensen; Annie Pedersen; Christian Blomstrand;
Kaj Blennow; Henrik Zetterberg; Katarina Jood; Christina Jern.

Methods

Definition of ischemic stroke and stroke subtyping

Ischemic stroke was defined as an acute onset of cerebral focal or global symptoms suggestive of stroke, no hemorrhage on brain CT or MRI scan, followed by a diagnostic work-up showing no signs of a non-vascular cause. All cases underwent ECG. Extracranial carotid and vertebral duplex ultrasound, CT angiography, magnetic resonance-angiography, catheter angiography, transcranial Doppler ultrasound, transthoracic and/or transesophageal echo-cardiography were performed when clinically indicated. Based on clinical presentation and results from the diagnostic work-up, cases were classified into ischemic stroke etiologic subtypes according to modified Trial of Org 10172 in Acute Stroke Treatment (TOAST) criteria.[1,2] In order to minimize interrater variability, the original TOAST criteria were refined according to a local protocol. Risk factors, other than atrial fibrillation and carotid stenosis (i.e. hypertension and diabetes mellitus), were not included in the protocol. Adjudication of subtypes was performed by two neurologists (K. J. and C. B.). Large artery atherosclerosis stroke was defined as either occlusive or significant stenosis (corresponding to > 50% diameter reduction according to the North American Symptomatic Carotid Endarterectomy Trial [NASCET] criteria [3]) of a clinically relevant precerebral or cerebral artery, presumably due to atherosclerosis, or a complex plaque (> 4 mm thick, ulcerated or mobile) in the aortic arch, and with potential causes of cardioembolism excluded. Small artery occlusion stroke was defined as a clinical lacunar syndrome with a relevant infarct of < 15 mm or normal CT/MRI in the absence of both a cardioembolic source and significant stenosis/occlusion due to atherosclerosis of an appropriate major brain artery. Cardioembolic stroke was defined as the presence of atrial fibrillation, sick sinus syndrome, myocardial infarction in the past four weeks, cardiac thrombus, infective endocarditis, atrial myxoma, prosthetic mitral or aortic valve, valvular vegetations, left ventricular akinetic segment, dilated cardiomyopathy, or patent foramen ovale in combination with either atrial septal aneurysm or deep venous thrombosis, and with significant stenosis/occlusion due to atherosclerosis of an appropriate precerebral or cerebral artery excluded. Other determined causes of stroke included those with arterial dissection, vasculitis,

hematologic disorders, monogenic syndromes and complications of cardiovascular procedures. Cryptogenic stroke was defined when no cause was identified despite an extensive evaluation. Undetermined stroke included cases for which more than one etiology was identified or when the evaluation was cursory.

Neurologic deficits at baseline

The maximum neurologic deficit within the first 7 days after the ischemic event was registered using the Scandinavian Stroke Scale (SSS) [4]. Since the National Institutes of Health Stroke Scale (NIHSS) is nowadays more commonly used than SSS, we estimated NIHSS scores, from SSS using a validated algorithm [5] to facilitate interpretation.

Vascular risk factors at baseline

Hypertension was defined by pharmacological treatment for hypertension and/or systolic blood pressure ≥ 160 mm Hg, and/or diastolic blood pressure ≥ 90mm Hg. Diabetes mellitus was defined by dietary or pharmacological treatment for diabetes and/or fasting plasma glucose ≥ 7.0 mmol/L, or fasting blood glucose ≥ 6.1 mmol/L. Hyperlipidemia was defined by pharmacological treatment for hyperlipidemia and/or total fasting serum cholesterol > 5.0 mmol/L, and/or low-density lipoprotein > 3.0 mmol/L. Smoking habit was coded as current versus never or former (smoking cessation at least one year before inclusion).

.

Tables and figures

Table S1. Brain regions on both sides where the presence of infarcts was registered.

**Frontal lobe**	
1.	Central frontal (below frontal horn)
2.	Central frontal (at the level of the frontal horn)
3.	Gyrus rectus & Gyrus orbitalis
4.	Gyrus cinguli (around frontal horns)
5.	Superior frontal gyri & Frontal pole
6.	Middle frontal gyri
7.	Inferior frontal gyri
8.	Gyrus precentralis
9.	Lobulus paracentralis
10.	Centrum semiovale (ACA supplied - anterior part)
11.	Centrum semiovale (MCA supplied – anterior part)
**Central gray & adjoining white matter**	
12.	Putamen
13.	Globus pallidus
14.	Caput nucleus caudatus
15.	Capsula interna anterior limb
16.	Capsula interna genu
17.	Capsula interna posterior limb
18.	Corona radiata
19.	Thalamus
20.	Hypothalamus
**Insula & adjoining gray & white matter**	
21.	Insulanear
22.	Capsula extrema, Capsula externa, Claustrum
**Temporal lobe**	
23.	Temporal central (at and below the level of the trigone)
24.	Superior temporal gyri
25.	Middle temporal gyri
26.	Inferior temporal gyri
27.	Transverse temporal gyri
28.	Corpus amygdaloideum
29.	Hippocampus/Parahippocampus
30.	Uncus
**Parietal lobe**	
31.	Gyrus postcentralis
32.	Lobulus parietalis superior
33.	Gyrus angularis
34.	Gyrus supramarginalis
35.	Precuneus
36.	Gyrus cinguli (posterior part)
37.	Central parietal (at and below the level of the lateral ventricle)
38.	Centrum semiovale (ACA supplied - posterior part)
39.	Centrum semiovale (MCA supplied - posterior part)
**Occipital lobe**	
40.	Central occipital
41.	Occipital pole & Lateral occipital gyri
42.	Visual cortex - Sulcus calcarinus-near
43.	Cuneus
**Corpus callosum**	
44.	Genu
45.	Truncus
46.	Splenium
**Infratentorial**	
47.	Mesencephalon
48.	Pons
49.	Medulla oblongata
50.	Cerebellum
**Other**	
51.	Trigonal area

ACA, anterior cerebral artery; MCA, middle cerebral artery.

Table S2 Patient characteristics at the index stroke for subjects with or without a history of previous stroke.. IQR = interquartile range; M = median; MRI = magnetic resonance imaging; NIHSS = National Institutes of Health Stroke Scale; SD = standard deviation; SSS = Scandinavian Stroke Scale; WMH = white matter hyperintensity. * $P < 0.05$; ** $P < 0.01$.

	**Subject with first-ever stroke** (n = 265)	**Subjects with previous stroke** (n = 51)
Age in years, mean (SD)	53 (11)	58 (7) **
Male sex, no. (%)	171 (65)	33 (65)
Hypertension, no. (%)	135 (51)	36 (71) *
Diabetes mellitus, no. (%)	41 (15)	13 (25)
Hyperlipidemia, no. (%)	177 (67)	38 (75)
Smoking, no. (%)	102 (38)	21 (41)
Subtype - Cryptogenic, no. (%)	100 (38)	10 (20) *
- Small artery occlusion, no. (%)	45 (17)	10 (20)
- Large artery atherosclerosis, no. (%)	32 (12)	14 (27) **
- Cardioembolic, no. (%)	29 (11)	9 (18)
- Cervical artery dissection, no. (%)	29 (11)	0 *
- Other determined, no. (%)	12 (5)	1 (2)
- Undetermined, no. (%)	18 (7)	7 (14)
SSS, M (IQR)	54 (42 to 57)	52 (46 to 55)
NIHSS (estimated), M (IQR)	2 (1 to 7)	3 (2 to 5)
Infarct location - Supratentorial cortical, no. (%)	108 (41)	21 (41)
- Supratentorial non cortical, no. (%)	74 (28)	13 (25)
- Infratentorial, no. (%)	43 (16)	3 (6)
- Supra and infratentorial, no. (%)	19 (7)	9 (18) *
- Undetermined, no. (%)	21 (8)	5 (10)
Small infarcts (< 2.5 cm^3), no. (%)	117 (44)	27 (53)
Fazekas - Deep WMHs > 1, no. (%)	39 (15)	18 (35) ***
- Periventricular WMHs > 1, no. (%)	24 (9)	14 (27) ***
Infarct vol. [cm^3], mean (SD)	26.4 (53.6)	36.0 (59.1)
WMH vol. [cm^3], mean (SD)	2.6 (6.2)	4.6 (8.0) *
Serum NfL [pg/ml], mean (SD)	181 (341)	155 (233)

Table S3 Correlations between sNfL concentrations and infarct/WMH volumes. (A) Pairwise Spearman's rank correlations obtained from the overall group. (B) Pairwise Spearman's rank correlations obtained in a subgroup analysis including only subject with small infarcts (< 2.5 cm3).. * P < 0.05; ** P < 0.01; *** P < 0.01.

A

	sNfL acute	sNfL 3 months	sNfL 7 years	Infarct vol. acute	WMH vol. acute	WMH vol. 7 years	Δ WMH vol.
sNfL acute	1	0.71***	0.13	0.66***	0.04	-0.05	0.03
sNfL 3 months	0.71***	1	0.14	0.70***	0.07	0.02	0.02
sNfL 7 years	0.13	0.14	1	-0.14	0.40***	0.49***	0.48***
Infarct vol. acute	0.66***	0.70***	-0.14	1	-0.17**	-0.21**	-0.16*
WMH vol. acute	0.04	0.07	0.40***	-0.17**	1	0.83***	0.67***
WMH vol. 7 years	-0.05	0.02	0.49***	-0.21**	0.83***	1	0.95***
Δ WMH vol.	-0.03	0.02	0.48***	-0.16*	0.67***	0.95***	1

B

	sNfL acute	sNfL 3 months	sNfL 7 years	Infarct vol. acute	WMH vol. acute	WMH vol. 7 years	Δ WMH vol.
sNfL acute	1	0.67***	0.51***	0.26**	0.23*	0.23*	0.21
sNfL 3 months	0.67***	1	0.47***	0.38***	0.28**	0.25*	0.21
sNfL 7 years	0.51***	0.47***	1	0.11	0.44***	0.55***	0.50***
Infarct vol. acute	0.26**	0.38***	0.11	1	-0.01	0.06	0.05
WMH vol. acute	0.23*	0.28**	0.44***	-0.01	1	0.86***	0.69***
WMH vol. 7 years	0.23*	0.25*	0.55***	0.06	0.86***	1	0.94***
Δ WMH vol.	0.21	0.21	0.50***	0.05	0.69***	0.94***	1

Figure S1. Correlations between sNfL concentrations and infarct/WMH volumes. Subjects in the acute phase were only included if blood sampling took place within the first 3 days.

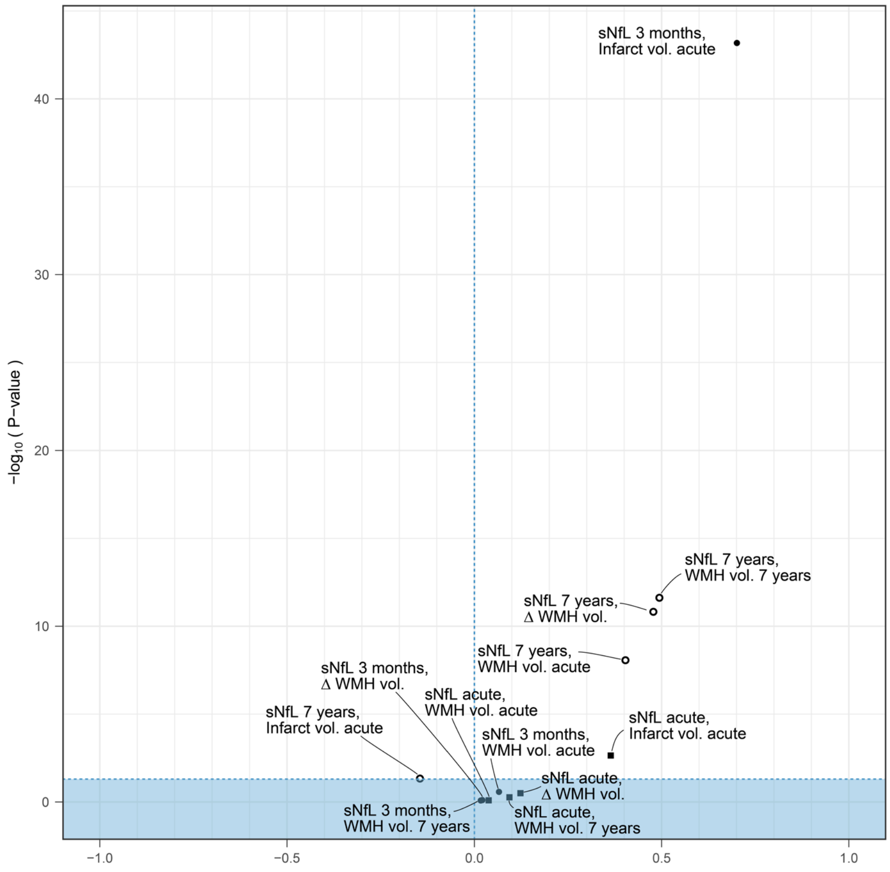

Pairwise Spearman's rank correlations. Shaded area represents P > 0.05. Δ = change from index stroke to 7-year follow-up; sNfL = serum neurofilament light chain protein; WMH = white matter hyperintensity.

Figure S2. Model of the increase in sNfL after ischemic stroke as a function of infarct volume and time since the stroke, with the observed data points from all subjects overlaid.

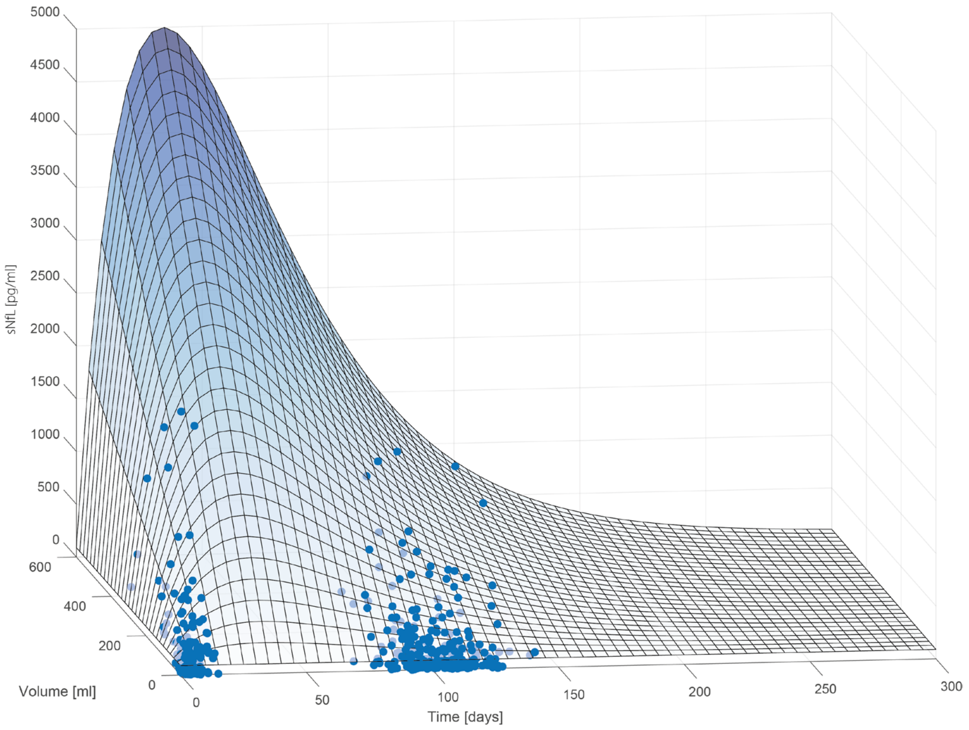

sNfL, serum neurofilament light chain protein.

References

1. Adams HP, Bendixen BH, Kappelle LJ, et al. Classification of subtype of acute ischemic stroke. Definitions for use in a multicenter clinical trial. TOAST. Trial of Org 10172 in Acute Stroke Treatment. *Stroke*. 1993;24(1):35-41.

2. Jood K, Ladenvall C, Rosengren A, Blomstrand C, Jern C. Family history in ischemic stroke before 70 years of age. *Stroke*. 2005;36(7):1383-1387.

3. North American Symptomatic Carotid Endarterectomy Trial Collaborators, Barnett HJM, Taylor DW, et al. Beneficial effect of carotid endarterectomy in symptomatic patients with high-grade carotid stenosis. *N Engl J Med*. 1991;325(7):445-453.

4. Scandinavian Stroke Study Group. Multicenter trial of hemodilution in ischemic stroke-background and study protocol. *Stroke*. 1985;16(5):885-890.

5. Gray LJ, Ali M, Lyden PD, Bath PMW. Interconversion of the National Institutes of Health Stroke Scale and Scandinavian Stroke Scale in acute stroke. *J Stroke Cerebrovasc Dis*. 2009;18(6):466-468.
